# Supplementary material for: Reconciling Mining with the Conservation of Cave Biodiversity: A Quantitative Baseline to Help Establish Conservation Priorities
Source: PLoS One. 2016 Dec 20;11(12):e0168348. doi: 10.1371/journal.pone.0168348 (PMC5173368; doi:10.1371/journal.pone.0168348)
Supplement: S1 Dataset — (ZIP) [file pone.0168348.s002.zip › Taxa/Serra Norte/SN_2013/N5S/Lista N5S-52.pdf]

CAVIDADE N5S-0052/0053

Classe	Ordem	Fam/Outros	Gên/Outros	Espécie	S-U	U-S	Zon.
Arachnida	Acari	Ixodidae	*Ixodes*	sp.1		X	A
Arachnida	Amblypygi	Phrynidae	*Heterophrynus*	*longicornis*	4	3	E/P/A
Arachnida	Araneae	Araneidae		jovem	X		P
Arachnida	Araneae	Barychelidae		jovem	1		E
Arachnida	Araneae	Caponiidae		sp.1	X	X	P/A
Arachnida	Araneae	Corinnidae	*Tupirinna*	sp.1	1	1	E
Arachnida	Araneae	Ctenidae		jovem	3	1	E/P
Arachnida	Araneae	Mysmenidae		jovem	X		E
Arachnida	Araneae	Ochyroceratidae	*Ochyrocera*	sp.1	X	X	A
Arachnida	Araneae	Ochyroceratidae	*Ochyrocera*	sp.3	X		P
Arachnida	Araneae	Ochyroceratidae	*Ochyrocera*	sp.4		X	A
Arachnida	Araneae	Oonopidae	*Gamasomorpha*	sp.1		X	E
Arachnida	Araneae	Oonopidae	*Neoxyphinus*	sp.2	X	X	P
Arachnida	Araneae	Pholcidae	*Mesabolivar*	*aurantiacus*	X	X	E/P
Arachnida	Araneae	Pholcidae	*Mesabolivar*	sp.1	X		E
Arachnida	Araneae	Segestriidae	*Ariadna*	sp.1	X		E
Arachnida	Araneae	Theraphosidae	aff. *Holothele*	sp.1	2	1	E
Arachnida	Araneae	Theridiidae	*Theridion*	sp.1		X	P
Arachnida	Araneae	Theridiidae	*Theridion*	sp.4		X	P
Arachnida	Araneae	Theridiosomatidae	*Plato*	sp.1	X	X	E/P/A
Arachnida	Opiliones	Cosmetidae	*Roquettea*	*singularis*	1		E
Arachnida	Opiliones	Escadabiidae		sp.1	X		P
Arachnida	Opiliones	Sclerosomatidae	*Prionostema*	sp.1	3	5	E
Arachnida	Opiliones	Stygnidae	*Protimesius*	*gracilis*	1		E
Arachnida	Pseudoscorpiones	Chthoniidae	*Pseudochthonius*	sp.1	X		E
Arachnida	Scorpiones	Buthidae	*Tityus*	sp.1		1	P
Chilopoda	Geophilomorpha	Geophilidae		sp.2		1	P
Chilopoda	Geophilomorpha	Geophilidae		sp.3	1		P
Chilopoda	Scolopendromorpha	Cryptopidae	*Cryptops*	jovem	1		P
Chilopoda	Scolopendromorpha	Scolopendridae	*Otostigmus*	sp.1		2	P
Chilopoda	Scolopendromorpha	Scolopocryptopidae	*Dinocryptops*	*miersii*		1	P
Chilopoda	Scutigeromorpha	Pselliodidae	*Sphendononema*	*guildingii*	1		E
Diplopoda	Polydesmida	Pyrgodesmidae		sp.1	X	X	P/A
Diplopoda	Polydesmida	Pyrgodesmidae		sp.4		X	A
Diplopoda	Spirostreptida	Pseudonannolenidae	*Pseudonannolene*	jovem		1	E
Entognatha	Collembola	Neelidae		sp.1		X	P
Entognatha	Collembola	Paronellidae		sp.1		X	E
Entognatha	Collembola	Sminthuroidea		sp.2		X	P
Entognatha	Diplura	Campodeidae		sp.1		X	E/P
Entognatha	Diplura	Japygidae		sp.3	X		E
Insecta	Blattodea	Blaberidae		sp.1	2		E
Insecta	Blattodea	Blattellidae		sp.1	4	2	E/P
Insecta	Blattodea	Blattidae		sp.5	2		P
Insecta	Blattodea	Polyphagidae		sp.1	5	1	P
Insecta	Coleoptera	Endomychidae		sp.1	X		E
Insecta	Coleoptera	Hydrophilidae		sp.2	X		P
Insecta	Coleoptera	Staphylinidae		sp.4	X		P
Insecta	Coleoptera	Staphylinidae		sp.6		X	E
Insecta	Diptera	Cecidomyiidae	Cecidomyiinae	sp.		X	E

Insecta	Diptera	Drosophilidae	*Drosophila*	*eleonorae*	X	X	E/A
Insecta	Diptera	Ephydridae		sp.		X	P
Insecta	Diptera	Phoridae	Phorinae	sp.	X	X	A
Insecta	Diptera	Psychodidae	Phlebotominae	sp.	X	X	E
Insecta	Diptera	Sciaridae		sp.	X		E
Insecta	Diptera	Tipulidae	Tipulinae	sp.	X	X	E
Insecta	Heteroptera	Dipsocoroidea		sp.	X	X	E/P/A
Insecta	Heteroptera	Reduviidae	*Zelurus*	sp.	3	7	E
Insecta	Heteroptera	Veliidae	*Paravelia*	sp.	X	X	P
Insecta	Homoptera	Cixiidae		sp.8	X	X	E
Insecta	Hymenoptera	Diapriidae		sp.1	X		A
Insecta	Hymenoptera	Formicidae	*Brachymyrmex*	sp.1	X		E
Insecta	Hymenoptera	Formicidae	*Camponotus*	sp.1	X		E
Insecta	Hymenoptera	Formicidae	*Camponotus*	sp.2		X	P
Insecta	Hymenoptera	Formicidae	*Paratrechina*	sp.1		X	E
Insecta	Hymenoptera	Formicidae	*Paratrechina*	sp.2	X		E
Insecta	Hymenoptera	Formicidae	*Pheidole*	sp.2	X	X	E/P
Insecta	Hymenoptera	Formicidae	*Pheidole*	sp.3	X		E
Insecta	Hymenoptera	Formicidae	*Pheidole*	sp.4	X		E
Insecta	Hymenoptera	Formicidae	*Solenopsis*	sp.1		X	E
Insecta	Isoptera	Termitidae	*Nasutitermes*	sp.	X	X	E/P/A
Insecta	Isoptera	Termitidae	*Subulitermes*	sp.	X	X	E
Insecta	Lepidoptera	Noctuoidea		sp.1		4	E/P
Insecta	Lepidoptera	Noctuoidea		sp.5	1		E
Insecta	Lepidoptera	Noctuoidea		sp.6		1	E
Insecta	Lepidoptera	Tineoidea		sp.1		X	P
Insecta	Lepidoptera	Tineoidea		sp.3	X	X	E/P
Insecta	Orthoptera	Gryllidae	Eneopterinae	sp.		1	E
Insecta	Orthoptera	Phalangopsidae	*Paraclodes*	sp.	1		E
Insecta	Orthoptera	Phalangopsidae	*Phalangopsis*	sp.	58	90	E/P/A
Insecta	Plecoptera	Gripopterygidae		jovem	1		E
Insecta	Psocoptera	Epipsocidae	*Mesepipsocus*	sp.1		X	E
Insecta	Psocoptera	Psyllipsocidae		jovem	X	X	P/A
Insecta	Thysanura	Nicoletiidae		jovem		X	E
Malacostraca	Isopoda	Armadillidae		sp.1	X	X	P
Malacostraca	Isopoda	Calabozoidea		sp.1	X		P
Malacostraca	Isopoda	Philosciidae		sp.2		X	P
Malacostraca	Isopoda	Scleropactidae	*Circoniscus*	*buckupi*	X	X	P/A
Turbellaria				sp.4	X		P/A
Amphibia	Anura			sp.	3	1	E/P
Mammalia	Chiroptera	Emballonuridae	*Peropteryx*	*kappleri*	3	6	E/P
Mammalia	Chiroptera	Furipteridae	*Furipterus*	*horrens*	3		P/A
Mammalia	Chiroptera	Phyllostomidae	*Desmodus*	*rotundus*	8		P/A
Mammalia	Rodentia	Cricetidae	*Rhipidomys*	sp.		2	A
